# Central Council for District Nursing in London

**Published:** 1920-11-13

**Authors:** 


### CENTRAL COUNCIL FOR DISTRICT NURSING IN LONDON.

It has been brought to the notice of the Central Council for District Nursing in London that there are considerable numbers of patients requiring skilled nursing who are neither, strictly speaking, the patients of the District Nursing organisations established for the nursing of the sick poor in their own homes nor are able to pay the full fees of a private nurse, or in some cases if able to pay full fees are yet unable to provide accommodation for a private nurse. These patients may be roughly classified as follows :—

1. Those who live in flats or other rooms without available additional accommodation for private nurses, even if able to pay full fees.
2. Patients of the professional classes unable to afford the ordinary fees of a private nurse.
3. Tradespeople and others not hitherto included in the work of the district nurse but urgently in need of some such assistance.

Efforts to meet these three classes of case seem so far to have been sporadic and unorganised; in London certain members of the Nurses' Co-operative have undertaken cases in Class 1, but from the reports of medical and other members of the Council it appears that such facilities either do not exist in sufficient number or, if existent, are not sufficiently known.

Some District Nursing Associations have endeavoured to meet the need in Class 3 in recent years, and most of them have agreed to allow their nurses to visit patients who can make some small payment, usually in the form of a donation. In the provinces a large number of Queen's Nursing Associations have drawn up scales of charges for visiting nurses for patients in not only classes 2 and 3 but also in class 1. In London this has not been largely done, but several of the associations are now considering the propriety of doing so, and would probably be willing to develop such a scheme. It appears clear that services rendered by District Nursing Associations should be limited to those who cannot pay the full fees necessary to put the scheme on to a profit-earning basis. It is clear, however, that a large number of the cases referred to fall below this level, and the Nursing Council consider that a scheme or schemes might usefully be drawn up for London districts.

For the cases in Class 1 it would appear that some such organisation as the Nurses' Co-operative is needed in various parts of London—possibly such organisation would devise a means by which this could be accomplished on a paying basis.

The following scale of fees might be considered :—3s. 6d. a visit, or £1 a week for daily visit; two daily visits at 2s. 6d. each, or 30s. a week for two daily visits.

The weekly *earnings* of a nurse with a due and manageable number of patients should thus be from 4 to 6 guineas a week at least.

Living together in small district communities, each nurse would contribute, say, 30s. a week to board and lodging and have remainder of the £3 3s. for personal expenses, travelling, dress, washing, etc.

The margin of earnings beyond £3 3s. could be reserved for insurance, sick fund, etc., and after these claims were satisfied any excess might be divided as a bonus at end of the year in some proportional manner agreed upon.

On the patients' side the economy would be :—

1. The saving from charges of a whole-time nurse, which at current rate is not less than £3 3s.
2. The saving of extra charges for washing and travelling expenses, now usually demanded.
3. The board of the nurse, sleeping accommodation—often difficult to provide—and accommodation during unemployed times.

For the cases in classes 2 and 3 the scale of fees would necessarily be lower, and the District Nursing Associations might consider some such scale as the following :—2s. 6d. or 3s. for a single visit; 12s. 6d. weekly for a daily visits; two daily visits, 15s. to £1; attendance on operation, 5s. to 10s.

